# Removal of H3K27me3 by JMJ Proteins Controls Plant Development and Environmental Responses in *Arabidopsis*

**DOI:** 10.3389/fpls.2021.687416

**Published:** 2021-06-17

**Authors:** Nobutoshi Yamaguchi

**Affiliations:** Division of Biological Science, Graduate School of Science and Technology, Nara Institute of Science and Technology, Ikoma, Japan

**Keywords:** *Arabidopsis*, development, demethylases, epigenetics, environmental response, JUMONJI, histone modification, H3K27me3

## Abstract

Trimethylation of histone H3 lysine 27 (H3K27me3) is a highly conserved repressive histone modification that signifies transcriptional repression in plants and animals. In *Arabidopsis thaliana*, the demethylation of H3K27 is regulated by a group of *JUMONJI DOMAIN-CONTANING PROTEIN* (*JMJ*) genes. Transcription of *JMJ* genes is spatiotemporally regulated during plant development and in response to the environment. Once *JMJ* genes are transcribed, recruitment of JMJs to target genes, followed by demethylation of H3K27, is critically important for the precise control of gene expression. JMJs function synergistically and antagonistically with transcription factors and/or other epigenetic regulators on chromatin. This review summarizes the latest advances in our understanding of *Arabidopsis* H3K27me3 demethylases that provide robust and flexible epigenetic regulation of gene expression to direct appropriate development and environmental responses in plants.

## Introduction

Chromatin is critically important for gene expression during plant development and in response to the environment (Eccleston et al., 2013; Bruneau et al., 2019). Chromatin is composed of genomic DNA, histones, and accessory proteins, with approximately 150 base pairs of DNA wrapped around each octameric histone protein complex (Vergara and Gutierrez, 2017; van Steensel and Furlong, 2019). Each histone protein consists of a structural core at the C terminus and an unstructured tail domain at the N terminus. The N-terminal flexible histone tails often possess extensive posttranslational modifications, such as acetylation, methylation, and ubiquitination on lysine residues, methylation and citrullination on arginine residues, and phosphorylation of serine, threonine, and tyrosine residues. These modifications cause epigenetic changes in chromatin and lead to changes in gene expression.

One chromatin modification, trimethylation of histone H3 lysine 27 (H3K27me3), mediates epigenetic silencing of gene expression (Xiao and Wagner, 2015; Xiao et al., 2016). In general, H3K27me3 marks occur within facultative heterochromatin, in which gene expression is repressed but can be activated in response to developmental or environmental cues. In animals and plants, H3K27me3 deposition and removal are mediated by specific enzymes termed “writers” and “erasers”, respectively. Polycomb repressive complex 2 (PRC2), a multisubunit epigenetic repressor complex, writes H3K27me3 marks associated with gene repression. By contrast, histone demethylases, such as the Jumonji C (JmjC)-containing eraser demethylases, can demethylate H3K27me3 and thereby counteract the action of writer methylases (Crevillén, 2020). Understanding the role of JmjC-containing demethylases is crucial to understanding the effects of H3K27me3 in plant development and environmental responses. Although PRC2 and its actions have been reasonably well characterized through decades of research, existing knowledge about H3K27me3 demethylases and demethylation is relatively limited. In the last decade, however, research on H3K27me3 removal in *Arabidopsis thaliana* (*Arabidopsis*) has made great progress. To date, five JMJ proteins have been identified as H3K27me3 demethylases: EARLY FLOWERING 6 (ELF6)/JUMONJI DOMAIN-CONTAINING PROTEIN11 (JMJ11), RELATIVE OF ELF6 (REF6)/JMJ12, JMJ13, JMJ30, and JMJ32 (Lu F. et al., 2011; Crevillén et al., 2014; Gan et al., 2014; Cui et al., 2016; Yan et al., 2018). Here, we summarize current understanding of a group of JmjC-containing demethylases of H3K27me3, with emphasis on the most recent advances in knowledge.

## H3K27 Demethylases Govern Many Processes in Plant Life

Upon sensing developmental or environmental cues, JMJ proteins make genomic regions accessible by removing repressive H3K27me3 marks to generate a legible genome that is specific to a particular cell type, developmental stage, or environmental condition. Functional analysis of loss-of-function *jmj* mutants in Arabidopsis has indicated that JMJ proteins make major contributions to developmentally or environmentally triggered transcriptional reprogramming events. REF6, ELF6, and JMJ13 make a broader contribution to plant growth and development than JMJ30 and JMJ32, which play more specific and redundant roles in environmental responses.

### H3K27 Demethylases Accumulate in Various Tissues

The divergence in the biological roles of H3K27me3 demethylases might be due to their different spatial and temporal expression patterns. The spatial distribution of REF6, ELF6, JMJ13, JMJ30, and JMJ32 proteins was examined by introducing constructs harboring their upstream and coding sequences fused with sequences encoding the β-glucuronidase (GUS) reporter into wild-type plants (Noh et al., 2004; Gan et al., 2014; Zheng et al., 2019). Among these five GUS reporters, JMJ30-GUS highly accumulated in various plant organs, such as leaves, roots, and flowers (Gan et al., 2014). REF6-GUS, JMJ13-GUS, and JMJ32-GUS show moderate accumulation in young leaves near the shoot apical meristem and in root tips but lower accumulation in the leaf vasculature (Noh et al., 2004; Gan et al., 2014; Zheng et al., 2019). By contrast, ELF6-GUS accumulates only in the distal part of young leaves. Current spatial expression data were obtained mainly by whole-mount GUS staining. Our understanding of JMJ accumulation is still limited largely to the organ level. Expression analysis derived from GUS staining may not be precise, due to diffusion of the enzyme outside the tissue, as compared with fluorescent protein–based experiments.

Transcriptome data from publicly available databases increase the understanding of demethylase function in Arabidopsis and allow functions to be inferred. Shoot apex–specific RNA sequencing (RNA-seq) and cell type–specific single-cell (sc) RNA-seq data revealed different expression patterns for the six *Arabidopsis* H3K27me3 demethylase genes (Winter et al., 2007; Ryu et al., 2019; Tian et al., 2019). In the shoot apical meristem, *JMJ30* is highly expressed, whereas the other genes are weakly expressed (Tian et al., 2019). Among eight different domains within the shoot apical meristem, *JMJ30* expression is higher in the *CLAVATA3* (*CLV3*) and *ARABIDOPSIS THALIANA MERISTEM LAYER 1* (*ATML1*) expression domains (Lu et al., 1996; Brand et al., 2002). Because *CLV3* and *ATML1* are specifically expressed in the central zone and layer 1, respectively, these observations suggest that *JMJ30* is also highly expressed in the center and/or epidermis of the shoot apical meristem. In the root, *JMJ13*, *ELF6*, *JMJ30*, and *RFF6* are expressed in a cell type–specific manner (Ryu et al., 2019). High *JMJ13* expression in the protoxylem suggests that it has a specific function in this tissue. These high-resolution differential expression patterns suggest that histone demethylation is tissue or cell type specific. Expression specificity at the cell-type or cellular levels needs to be characterized in detail to further our understanding of when and where JMJ proteins work.

Although epigenetic regulation is thought to be important in responses to environmental stimuli, few reports have described the relationship between environmental stress and the induction of *JMJ* genes. *JMJ30* expression is further enhanced by the stress hormone abscisic acid (ABA) and by salt stress, drought stress, and heat stress compared to control conditions (Qian et al., 2015; Wu et al., 2019a; Yamaguchi et al., 2020). ABA treatment triggers a rapid increase in JMJ30 protein levels but does not change the area of JMJ30 expression, based on whole-mount GUS staining (Wu et al., 2019a). The expression of *JMJ13* is affected by light and temperature conditions, according to GUS expression data (Zheng et al., 2019). *REF6* expression is induced by long-term heat exposure (Liu et al., 2019). To date, no effects of environmental stress on the regulation of *ELF6* and *JMJ32* expression have been reported. Bulk transcriptome datasets also largely support these results (Qian et al., 2015). Expression specificity and subcellar localization of JMJ proteins in response to environmental stimuli should also be addressed with higher resolution in the future.

### Mutant Phenotypes and Key Targets of H3K27 Demethylases

The seeds of the *ref6* mutant germinate later than wild type (Li et al., 2016; Chen et al., 2020). REF6 induces two key genes for ABA catabolism, *CYP707A1* and *CYP707A3*, through removal of H3K27me3. *CYP707A1* and *CYP707A2* encode ABA 8′-hydeoxylases and play key roles in reducing ABA levels (Okamoto et al., 2006). Overexpression of *CYP707A1* by cauliflower mosaic virus (CaMV) 35S promoter rescues the dormancy phenotype of the *ref6* mutant (Chen et al., 2020). The *jmj30 jmj32* double mutant, by contrast, shows no difference in seed dormancy phenotype from wild type (Wu et al., 2019a).

Under normal growth conditions, *ref6* and *elf6* mutants have similar leaf phenotypes that include reduced petiole length, which is characteristic of brassinosteroid (BR)-defective mutants (Yu et al., 2008). A shorter leaf blade is seen in *ref6* but not *elf6* plants, suggesting that the REF6 and ELF6 proteins have tissue-specific roles. The *ref6* mutation further enhances the phenotype of a BR-deficient mutant. In the *ref6 elf6* double mutant, expression of BR-regulated genes, such as *TOUCH 4* (*TCH4*), is reduced. Later in leaf development, *ref6* delays chlorophyll degradation (Wang et al., 2019) and REF6 promotes general leaf senescence by directly activating senescence-related genes, including *ETHYLENE INSENSITIVE 2* (*EIN2*), *OLEOSIN 1* (*ORE1*), and *NONYELLOWING* genes (*NYE*s). The *jmj13* single mutant does not display detectable abnormalities in leaf phenotype (Zheng et al., 2019), but the *ref6 elf6 jmj13* triple mutant has shorter petioles than *ref6 elf6*, suggesting that REF6, ELF6, and JMJ13 are essential developmental regulators (Yan et al., 2018). The *jmj30 jmj32* double mutant, by contrast, shows no difference in leaf phenotype from wild type (Yamaguchi et al., 2020).

All five Arabidopsis H3K27me3 demethylases regulate flowering time, but in distinct fashions. *REF6* and *ELF6* were originally identified on the basis of their influence on flowering-time phenotypes: under long-day conditions, *ref6* mutants are late flowering and *elf6* and *jmj13* mutants are early flowering (Noh et al., 2004; Zheng et al., 2019). REF6 directly induces floral activator genes, such as *SUPPRESSOR OF OVEREXPRESSION OF CONSTANS 1* (*SOC1*) and *FRUITFULL* (*FUL*) (Hou et al., 2014; Hyun et al., 2016). ELF6 binds to the regulatory region of the floral repressor gene *FLOWERING LOCUS C* (*FLC*) for transcriptional activation (Yang et al., 2016). In *jmj13* mutants, the floral repressor gene *SHORT VEGETATIVE PHASE* (*SVP*) is downregulated. By contrast, flowering-time defects in *jmj30 jmj32* are observed only at high ambient temperatures but not under long-day conditions (Gan et al., 2014; Yan et al., 2014). Thus, the H3K27me3 demethylases *REF6*, *ELF6*, *JMJ13*, *JMJ30*, and *JMJ32* show differences as well as similarities in how they influence flowering time.

Differences are also observed between the phenotypes of *elf6* and *jmj13* during flower development (Keyzor et al., 2021). In wild type and the *ref6* mutant, the initial one or two flowers do not undergo self-pollination and form very short fruits without seeds; *elf6* plants display increased self-fertility and consistent fruit production. Conversely, the *jmj13* mutant shows reduced fertility and gives rise to aborted fruits up to the eighth flower on the primary inflorescence. *JASMONATE-ZIM-DOMAIN PROTEIN 7* (*JAZ7*), *SMALL AUXIN UP RNA 26* (*SAUR26*) and *ARABINOGALACTAN PROTEINs* (*AGPs*) are downregulated in *jmj13* buds. No defects in floral developmental have been reported for *jmj30* and *jmj32* mutants.

The functions of JMJ30 and JMJ32 appear to be relatively distinct from those of REF6, ELF6, and JMJ13. *jmj30* mutants show a circadian phenotype (Jones et al., 2010), and the *JMJ30* gene was originally identified due to its co-expression with *TIMING OF CAB1 EXPRESSION 1* (*TOC1*). Consistent with the circadian oscillation in *JMJ30* expression, circadian rhythms in reporter-gene activity in *jmj30* mutants are significantly shorter than those in wild type. *JMJ30* and *TOC1* interact genetically to promote the expression of *CIRCADIAN CLOCK ASSOCIATED 1* (*CCA1*) and *LATE ELONGATED HYPOCOTYL* (*LHY*). By contrast, no circadian oscillation in *JMJ32* expression is observed (Lu S. X. et al., 2011), suggesting that *JMJ30* and *JMJ32* are regulated by distinct mechanisms.

*jmj30* mutants also feature phenotypes that are dependent on environmental conditions. Callus formation induced by incubating leaf explants on callus-inducing medium is reduced in *jmj30* mutants. JMJ30 promotes the expression of *LATERAL ORGAN BOUNDARIES DOMAIN 16* (*LBD16*) and *LBD29* to establish root primordium–like unorganized cell masses (Lee et al., 2018). Furthermore, stress hormone–induced growth arrest is compromised in *jmj30 jmj32* double mutants (Wu et al., 2019a, b, 2020), and JMJ30 directly activates *SNF1-RELATED PROTEIN KINASE 2.8* (*SnRK2.8*) and *BRASSINAZOLE RESISTANT1* (*BZR1*) to maintain a balance between stress responses and growth. Acquired thermotolerance is also reduced in *jmj30 jmj32 ref6 elf6* quadruple mutants (Yamaguchi et al., 2020). JMJ30 binds to *HEAT SHOCK PROTEIN 17.6C* (*HSP17.6C*) and *HSP22* and activates their transcription in response to heat.

Although the interactions between JMJ proteins and downstream targets is regulated in a spatiotemporal manner, it is not yet known how exactly JMJ proteins lead to H3K27me3 removal. Most phenotyping has been conducted in knock-out or knock-down mutants, while mutant rescue by expressing downstream targets has used CaMV35S-based overexpression lines (Wu et al., 2019a; Chen et al., 2020; Yamaguchi et al., 2020). To assess the precise roles of JMJ during plant development and environmental responses, conditional *jmj* mutants should be employed. Furthermore, organ-, tissue-, or cell type–specific phenotypic rescues using appropriate promoters are required to understand when and where JMJ proteins function.

## Protein Structure and Chromatin-Targeting Mechanisms of H3K27 Demethylases

Phylogenetic analysis of JmjC-containing demethylases defined 14 subfamilies and identified more than 10 members in land plants. Green algae such as *Chlamydomonas* and *Volvox* include only two members of this family, implying that the functions of JMJ proteins may have been important for plant adaptation to land (Qian et al., 2015). Arabidopsis contains 21 JMJ proteins (Lu et al., 2008). Although not all family members have been fully characterized, they include putative H3K9me3-, H3K36me3-, H3K4me3-, and H3K27me3-specific demethylases. Additional H3K27me3 demethylases may also exist. ELF6 and REF6 show highest sequence similarities to the H3K9me3- and H3K36me3-specific KMD4 demethylases. The precise functions of the remaining JMJ proteins need to be carefully examined in a manner that is unbiased by sequence similarity.

**FIGURE 1:**
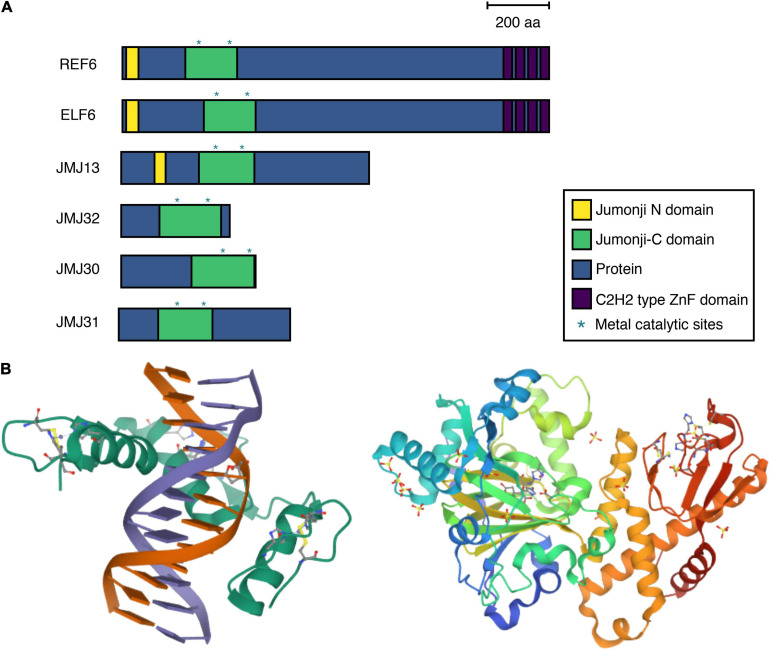
Conserved motifs, and three-dimensional structure of H3K27me3 demethylases in *Arabidopsis thaliana*. **(A)** The domain structures of Jumonji domain–containing proteins (JMJs) in *A. thaliana*. The positions of the Jumonji N domain, Jumonji C domain, protein, and C2H2-type zinc-finger (ZnF) domains are indicated in yellow, green, blue, and purple, respectively; metal catalytic sites are indicated with asterisks. Scale bar = 200 amino acids. **(B)** Crystal structure of JMJ proteins. *Left* REF6 ZnF and *NAC004*-mC3 double-stranded (ds) DNA. Ribbon representation of REF6-DNA structure. REF6 protein is shown in green, while dsDNA is shown in orange and purple. α-helices and β-sheets are represented by spiral ribbons and green arrows, respectively. A few residues engage Zn^2+^ ion. *Right* The JMJ13 catalytic domain in complex with AKG. Ribbon representation of JMJ13 structure. Blue and green ribbons represent JMJ domains. Orange and red ribbons show helical and ZnF domains, respectively. The data were obtained from the Protein Data Bank (https://biorender.com).

**FIGURE 2:**
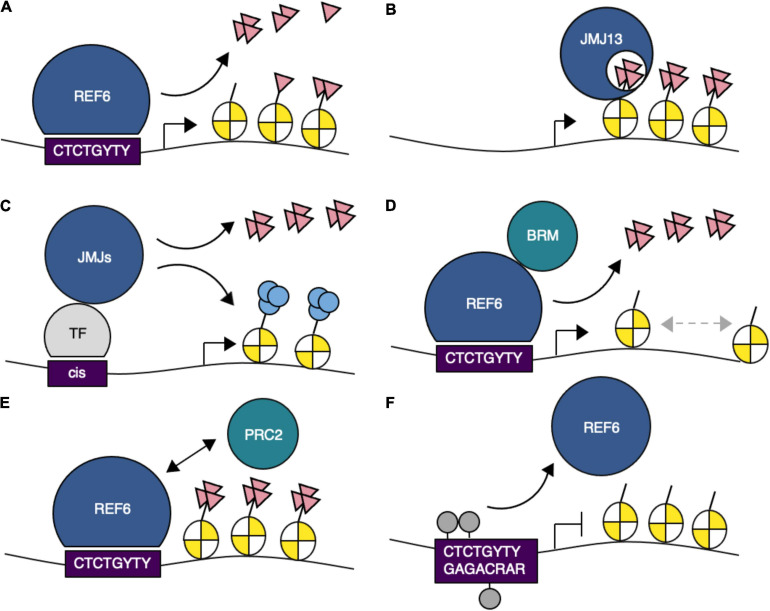
Schematic representation of the function of H3K27me3 demethylases in gene regulation. **(A)** REF6 demethylates H3K27me1/2/3 via recognition of CTCTGYTY (where Y is C or T) DNA motifs. **(B)** JMJ13 recognizes H3K27me3 marks. **(C)** JMJ proteins interact with transcription factors and are recruited to their target sites. **(D)** REF6 interacts with BRAHMA (BRM) and mediates nucleosome positioning. **(E)** REF6 prevents the uncontrolled spreading of PRC2-mediated chromatin silencing. **(F)** DNA methylation at CTCTGYTY motifs prevents REF6 targeting. H3K27me3, pink triangles; H3K9ac, light blue circles; DNA methylation, gray circles.

The ELF6, REF6, and JMJ13 proteins belong to the plant-specific KMD4 subfamily, which is present in land plants but not in green algae (Lu et al., 2008; Qian et al., 2015). REF6 contains JmjN, JmjC, and C2H2-type zinc-finger (ZnF) domains (Figure 1A). The REF6 protein characteristically possesses four tandem repeats of the ZnF domain. These domains are essential for REF6 function, as complementation of the *ref6* mutant through the introduction of REF6 without a ZnF domain fails to rescue the *ref6* mutant phenotype. Some histone demethylases, such as KDM2, interact with chromatin via direct binding to DNA through ZnF domains. ZnF is one of the largest class of DNA-binding domains. Consistent with this, REF6 functions as a DNA sequence–specific H3K27me3 demethylase. Genome-wide REF6 binding studies and crystal structure analysis revealed that the ZnF domains of REF6 recognize the CTCTGYTY DNA motif for H3K27me3 removal (Figures 1B, 2A) (Cui et al., 2016; Li et al., 2016; Tian et al., 2020). The ZnF domains of REF6 complex with *NAC004* double-stranded (ds) DNA by forming a half-cross-braced structure (Figure 1B). Interactions at the interface between REF6 and dsDNA, such as hydrogen bonds, electrostatic interactions, and hydrophobic interactions, strengthen their binding. dsDNA binding induces profound conformation changes (Tian et al., 2020). Conformational plasticity of DNA allows REF6 to recognize diverse target genes.

EARLY FLOWERING 6 is the closest homolog of REF6. Those two proteins share a high sequence similarity. How ELF6 recognizes DNA is currently unknown. Because the mutant phenotypes of *elf6* and *ref6* differ, the recognition motifs or mechanisms might also differ between REF6 and ELF; a recent study showed that REF6 and ELF6 play distinct roles in H3K27me3 and H3K27me1 homeostasis (Antunez-Sanchez et al., 2020). ELF6 regulates a small subset of genes compared to REF6. This could be due to less protein structural plasticity of ELF6 and/or a difference in DNA-binding affinity. Further analysis, such as determination of crystal structures of ELF6-DNA complexes, is required to reveal the structural basis for the epigenetic modification recognition.

Although JMJ13, like REF6, belongs to the KMD4 subfamily and functions to remove H3K27me3 *in vitro* and *in vivo*, it possesses a different DNA-recognition mechanism. JMJ13 does not contain ZnF domains at the C terminus (Figure 1A); instead, its catalytic domain (JMJ13CD) contains Jmj and helical domains, as well as a unique C4HCHC-type ZnF domain. Crystal structure analysis using JMJ13CD revealed that JMJ13 recognizes the H3K27me3 peptide and functions as a reader of the histone modification state (Figures 1B, 2B) (Zheng et al., 2019). The interactions between JMJ13 and the H3K27me3 peptide are restricted to the region between H3R26 and H3P30. Because other JMJ proteins are predicted to possess different putative ZnF domains, such as the C5HC2-type, the recognition of histone modifications by ZnF domains located within the JmjC domain might also occur for other JMJ proteins.

JMJ30 and JMJ32, as well as their close homolog JMJ31, belong to the JmjC-domain-only group. Consistent with their domain structure, two clades of protein homology are identified by phylogenetic analysis: one contains ELF6, REF6, and JMJ13 and the other contains JMJ30, JMJ31, and JMJ32 (Lu et al., 2008; Qian et al., 2015). Although the function and regulation of JMJ30 and JMJ32 are relatively well characterized, nothing is known about their three-dimensional protein structure or DNA–histone recognition mechanisms. As is often observed for REF6 and ELF6 recruitment (Figure 2C), JMJ30 physically interacts with tissue-specific transcription factors, such as EARLY FLOWERING MYB PROTEIN (EFM) and AUXIN RESPONSE FACTORs (ARFs) (Yan et al., 2014; Lee et al., 2018). Furthermore, JMJ30 activity affects H3K9me3 and H3K36me3 in addition to H3K27me3. Further experiments, such as JMJ30 chromatin immunoprecipitation and deep sequencing (ChIP-seq), are required to precisely understand their biochemical functions.

Genome-wide JMJ protein binding and histone modification data were obtained by ChIP-seq. Since ChIP-seq assays require large numbers of input cells/tissues, whole plants are often used for the assays. Hence, spatial information is completely lost. Recently, low-input binding tests in plants, such as CUT&Tag, CUT&RUN, nCUT&Tag, and ChIL, have been developed to study interactions between DNA and proteins using low-input samples or single live cells (Zheng and Gehring, 2019; Sakamoto et al., 2020; Tao et al., 2020; Ouyang et al., 2021). By combining these with cell sorting or laser microdissection techniques, cell type–specific JMJ protein binding and histone modification data can be obtained in the future. These analyses may contribute to our understanding of the precise spatiotemporal regulation of H3K27me3 demethylation.

## Interactions Between H3K27 Demethylases and Other Factors on Chromatin

Genome-wide binding analysis coupled with immunoprecipitation and mass spectrometry (IP-MS) identified an interaction between REF6 and the SWI/SNF-type chromatin remodeling ATPase BRAHMA (BRM) on chromatin *in vivo* (Li et al., 2016). REF6 and BRM bind to many common genomic loci that contain CTCTGYTY motifs. Recruitment of BRM to target loci is dependent on REF6 function, but REF6 does not require BRM activity for its own targeting. Thus, REF6 directly binds to chromatin containing the CTCTGYTY motifs and subsequently recruits BRM to activate targets, potentially through changes in nucleosome position (Figure 2D).

An antagonistic role between REF6 or BRM and PRC2 at their target loci has been demonstrated (Bezhani et al., 2007; Lu F. et al., 2011; Wu et al., 2012; Li et al., 2015). Antagonism is often mediated by competitive binding at the same sites on chromatin (Zhu et al., 2020). However, PRC2 preferentially binds to different motifs, such as the telobox and GAGA motifs (Hecker et al., 2015; Xiao et al., 2017; Zhou et al., 2018), suggesting that competitive antagonism is unlikely to occur between REF6/BRM and PRC2; moreover, the binding patterns of PRC2 and REF6 do not overlap. REF6 is localized to the boundaries of H3K27me3 regions, which are covered by PRC2 (Yan et al., 2018) (Figure 2E). The spreading of H3K27me3 observed in *ref6 elf6 jmj13* triple mutants indicates that the function of REF6 binding inhibits the spreading of H3K27me3, but how ELF6 and JMJ13 contribute to preventing this spreading remains unclear.

Recognition of dsDNA by REF6 not only relies on DNA sequence but also is affected by DNA methylation and sequence-dependent conformations of DNA (Qiu et al., 2019). REF6 preferentially binds to hypomethylated CTCTGYTY motifs. Methylation of CHG within the motif attenuates REF6-binding affinity (Figure 2F), and the minor groove width of each nucleotide in the structure of the complex differs considerably. This difference affects recognition of the CTCTGYTY motifs by REF6 and its binding affinity in *CUP-SHAPED COTYLEDON 1* (*CUC1*) and *CUC2* (Tian et al., 2020). The possibility that factors other than DNA sequence may contribute to REF6 binding affinity is supported by the fact that REF6 recognizes only 15% of the CTCTGYTY motifs in the Arabidopsis genome.

Protein–protein interaction between JMJ proteins and other transcription/chromatin factors are critical for H3K27me3 removal. However, conclusive *in vivo* evidence of when and where exactly those factors interact each other is lacking. Innovative *in vivo* imaging techniques are used to understand plant development and environmental responses through spatiotemporal regulation of gene expression (Abe et al., 2019; Hirakawa et al., 2019). Application of these techniques in H3K27me3 demethylase research to reveal the distribution of JMJ protein complexes will provide new insights into the spatiotemporal regulation of H3K27me3 removal.

## Conclusion and Perspectives

Flexible and robust gene expression during plant development and in response to the environment is primarily controlled by epigenetic regulation. In the past 5 years, plant epigenetic research on demethylases using transcriptome, epigenome, and crystal structure analyses has revealed the importance of H3K27me3. In *Arabidopsis thaliana*, the demethylation of H3K27 is regulated by a group of *JUMONJI DOMAIN-CONTANING PROTEIN* (*JMJ*) genes. *JMJ30* expression is high in various organs and is further boosted in response to environmental cues. On the other hand, the expression levels of *REF6*, *ELF6*, *JMJ13*, and *JMJ32* is moderate. *REF6* and *JMJ13* expression is also affected by environmental cues. These H3K27me3 demethylases bind to chromatin through generic or sequence-specific targeting mechanisms: direct binding to DNA via a ZnF domain, direct recognition of H3K27me3, or indirect binding through interactions with transcription factors. DNA methylation and minor groove width also fine-tune the binding affinity of these H3K27me3 demethylases. The targeting and occupancy of the histone demethylases on chromatin antagonize PRC2-mediated H3K27me3 deposition, and the removal of histone demethylases and prevention of their uncontrolled spread determine the shape of the H3K27me3 peak. Subsequently, H3K27me3 demethylases recruit a chromatin remodeler to activate gene transcription. One major limitation in current epigenome research is the scarcity of spatial information concerning the binding of epigenetic regulators and the nature of epigenetic modifications and co-factors. Furthermore, when and where target expression by H3K27me3 demethylases is mediated are poorly understood. Both binding patterns and DNA–protein structures and/or co-factors might vary among cells, tissues, and organs. In addition, growth conditions affect the epigenomic dynamics among individual plants. Therefore, specific genomic profiles obtained from plants grown under different conditions are needed to understand the specific roles of H3K27me3 demethylases during plant development and responses to the environment.

## Author Contributions

NY conceptualization and writing the manuscript.

## Conflict of Interest

The author declares that the research was conducted in the absence of any commercial or financial relationships that could be construed as a potential conflict of interest.
